# Candidate Soluble Immune Mediators in Young Women with High-Risk Human Papillomavirus Infection: High Expression of Chemokines Promoting Angiogenesis and Cell Proliferation

**DOI:** 10.1371/journal.pone.0151851

**Published:** 2016-03-18

**Authors:** Nunzia Zanotta, Maria Lina Tornesello, Clorinda Annunziata, Giovanni Stellato, Franco Maria Buonaguro, Manola Comar

**Affiliations:** 1 Institute for Maternal and Child Health – IRCCS “Burlo Garofolo”, Trieste, Italy; 2 Molecular Biology and Viral Oncology Division, Istituto Nazionale Tumori IRCCS – “Fond. Pascale,” Napoli, Italy; 3 Gynecology Oncology Division, Istituto Nazionale Tumori IRCCS – “Fond. Pascale,” Napoli, Italy; 4 Department of Medical Sciences, University of Trieste, Trieste, Italy; Rudjer Boskovic Institute, CROATIA

## Abstract

**Background:**

The causal interpretation of cervical immune response to Human Papillomavirus (HPV) infection is complex and poorly characterized mainly due to the delicate balance that exists between viral infection, increase of inflammatory cytokines and host risk factors. This study aims to explore the significance of cervical immune mediators associated to cell survival, angiogenesis and interaction with immune response, in predicting the risk to develop HPV-related intraepithelial lesions.

**Methods:**

A panel of 48 cytokines and growth factors were explored in a selected cohort of 168 immunocompetent women including 88 diagnosed with low (LSIL) or high (HSIL) squamous intraepithelial lesions of the cervix and 80 with normal cervical cytology (NIL). HPV genotyping was performed by Linear Array HPV test and the soluble concentration of 48 immune molecules was analyzed using the Bio-Plex platform.

**Results:**

The prevalence of single HR-HPV infection was 30% in NIL and 100% in LSIL and HSIL women. The expression of 13 cytokines, including interleukins IL-6, IL-3, IL-12p40, IL-12p70, IL-16, IL-18, LIF, of chemokines CCL7 (MCP-3), CXCL9 (MIG), CXCL12 (SDF-1α) and of the tropic factors VEGF, G-CSF, M-CSF were significantly associated with the presence of infection, with levels being higher in women with precancerous lesions compared to NIL HPV negative women. Only the growth factor GM-CSF was positively associated with the cytological abnormalities.

**Conclusions:**

The ability of HR-HPV to escape from innate immune recognition and to orchestrate the production of specific inflammatory and growth factors, involved in early inflammatory response and in the cell-proliferating phase of intraepithelial damage, was documented in women before the development of cervical lesions.

## Introduction

Cervical cancer (CC) is the third most common cancer in women worldwide accounting for 13% of all female cancers in developed countries. Persistent infection with High Risk Human Papillomaviruses (HR-HPVs) is considered the major cause of CC [1–4]. Most sexually active women will have an HPV infection at some time in their life, with or without low intraepithelial lesions (LSIL), which will be spontaneously cleared by the immune system. However, in a small proportion of women the virus is able to establish a persistent infection, probably due to the synergistic effect of suboptimal host-dependent immune response and HPV-induced immunological changes, and may cause a different clinical outcome [5–7]. Escape from innate immune recognition seems to be the hallmark of HPV pathogenesis. Failure to develop an effective cell-mediated immune response results in persistent infection and increased risk of malignant transformation of the cervical cells [8,9]. Central to this achievement is the ability of HPV to compromise the innate immune signaling pathway in the infected keratinocytes of the basal layer of the cervical epithelium [7,8]. During normal HPV replication, which takes place within maturing squamous epithelium, HPV remains hidden from the host immune system, suggesting that the opportunity for HPV to induce or modulate specific immune responses is limited [10]. The immune evasion mechanism exerted by HPV as a necessary mechanism to favour viral infection results in the impairment of the antigen processing and presentation machinery, the down-regulation of adherence molecules and of cytokine expression [11–18]. In the cervical tissue, the cells of the innate immune system represent the first barrier against HPV infection and replication, inducing the secretion of pro-inflammatory cytokines, such as interleukins IL-1b, IL-6, IL-8, IL-12, tumor necrosis factor (TNF-α) and interferons (INFs) which are essential for the activation of the adaptive immune response to local injury. Conversely, an imbalance in this pro-inflammatory network, almost from the very first phases of the infectious cycle, can positively affect cell transformation [13,15,16,] [19–22]. The non-structural HPV oncoproteins E6 and E7, have been shown to induce anti-inflammatory and immune suppression mechanisms [23]. The over-expression of the E6 and E7 oncogenes has been associated with the deregulation of cytokines and corresponding receptors, resulting in the impairment of cytotoxic effectors in the cervical lesions [24]. Specifically, multiple lines of evidence suggested that HPV oncoproteins can directly affect the response to IFN-α and IFN-γ signaling and the expression of key intermediate transcription control factors, such as Tyk2 kinase, STAT1 and STAT2 [25–29]. The E6 and E7-dependent reduction in keratinocyte secretion of some potent chemoattractant, including macrophage inflammatory protein 3 alpha (CCL20, MIP-3α) and IL-8, and the down-regulation of the Toll-like receptor TLR9, which play an important role in virus DNA clearance, seem to favor long-term HPV persistence and the establishment of neoplasia [30–33]. Moreover, it has been suggested that cervical cancer progression could be linked both to an undesirable Th1- to Th2-cytokine type shift and to an increased production of interleukin IL-10 triggered by two E7-derived epitopes [34,35]. The “in vivo” characterization of the cervical microenvironment during HPV infection and before cell damage, represents a crucial point to broaden current knowledge on the contribution of host-dependent factors in HR-HPV diseases [11,12,18].

However, the”in vivo” causal interpretation of mucosal cytokines expression is difficult, mainly due to the delicate balance between pro-inflammatory and anti-inflammatory cytokines which is affected by HPV and by host risk co-factors that can influence the progression of carcinogenesis.

The aim of this study was to analyze the”in vivo” cervical cytokines profile of HR-HPV infections in the absence of additional cervical cancer co-factors that may drive the cytokine network. Th1/Th2, T-reg, Th17 cells, Th9 cytokines and growth factors linked to cell survival and angiogenesis, were studied in cervical exfoliates positive for single genotype of HPV from a selected cohort of young women living in geographical regions at average incidence for cervical cancer, and negative for sexually transmitted diseases (STD) other than HPV.

## Materials and Methods

### Ethical statement

The study was approved by the Institutional Scientific Board of the Institute for Maternal and Child Health—IRCCS “Burlo Garofolo” of Trieste, Italy and written informed consent was obtained from each participant in accordance with the principles outlined in the Declaration of Helsinki.

### Patients and Samples Collection

Cervical cells and cervical-vaginal fluid samples were collected from a selected group of women recruited as outpatients at the Gynaecology Department Service, to detect HPV and evaluate cytokines concentration. Baseline eligibility criteria of enrolled women included: 20–35 years of age, Caucasian origin, no current use of tobacco, alcohol or contraceptives, no hospitalization in the preceding 6 months, no chronic systemic medication, no hematological evidence of autoimmune disorders (negative for antibody (Ab) anti-nucleus and Ab anti-DNA), positive Th1/Th2 response in previous diagnosed infections, not HPV vaccinated, negative for bacterial vaginosis and STD including Chlamydia Trachomatis, Neisseria gonorrhea, Trichomonas vaginalis, Micoplasma Hominis/Genitalium infections, “healthy” on the day of enrollment.

Cervical cells were obtained using a cervex brush device (Rovers Medical Devices B.V. The Netherlands), reaching the endocervical canal and touching both the ectocervical area and the transformation zone (T-zone), and transferred in 500 μL of sterile water. The cervico-vaginal fluid was collected using 3 mL of sterile water in a sterile plastic transfer pipette, vigorously flushed several times over the cervix. The material was collected with the same pipette and placed in a test tube on ice. Aliquots (0.1 mL) were prepared immediately and stored at -80°C. Cytopathological analyses were performed according to the Bethesda System 2001 [36].

### DNA Extraction, Sexual Transmitted Microorganisms Detection

DNA was extracted from cervical cell samples using the QIAamp DNA Blood miniKit (Qiagen, GmbH, Germany) as indicated by the supplier, and stored at -80°C. The presence of C. trachomatis, Mycoplasma (Hominis/Genitalium), Neisseria gonorrhoeae and Trichomonas vaginalis DNA was detected by Real Time PCR (RT-PCR) using a commercial kit (RealLine Pathogen Diagnostic Kits, Bioron Diagnostics). The amplification and PCR product detection were performed by CFX96^™^ (Bio-Rad, USA). Briefly, 25 μL of diluted Master Mix were added to each 0,2 mL tube, then 25 μL (50 μL for Trichomonas and Neisseria) of corresponding isolated DNA solution were added to each tube. The temperature profile was: 2 min at 50°C, 2 min at 94°C, 10sec at 94°C, and 40 sec at 60°C, for 50 cycles.

### Linear Array HPV Genotyping Test (LA)

HPV DNA was extracted from 500 μL of each exfoliated cervical sample, eluted in a total volume of 100 μL using a commercial kit (High Pure PCR Template preparation Kit, Roche Applied Science, Mannheim, Germany) as indicated by the supplier.

HPV detection and genotyping was performed by Linear Array HPV genotyping test (LA) (Roche Diagnostics) using biotinylated primers to define a sequence of 450 nucleotides within the polymorphic L1 region of the HPV genome. The pool of HPV primers is designed to amplify HPV DNA from 37 genotypes, including 20 HR/pHR-HPV types (16, 26, 18, 31, 33, 35, 39, 45, 51, 52, 53, 56, 58, 59, 66, 67, 68, 70, 73 and 82), and to amplify the β-globin human gene as a genomic DNA control. The LA assay was used manually as described by the manufacturer. At least one replicate of the linear array HPV positive (HPV-16) and negative controls was processed within each run.

### Cytokines analysis

The main outcome of this study was the quantification of cytokines and growth factors concentrations, based on magnetic bead multiplex immunoassays (Bio-Plex, BIO-RAD Laboratories, Milano, Italy). Luminex multiplex panel technology was used for simultaneous measurement of a panel of 48 analytes including cytokines, chemokines and growth factors (Table 1).

**Table 1:** Cytokines, chemokines and trophic factors detected by Luminex multiplex panel technology.

	Cytokines	Chemokines	Trophic factors
**21-plex (BioRad)**	IL-1α; IL-2Rα; IL-3; IL-12 (p40); IL-16; IL-18; IFN-α2; LIF; MIF; SCF; TNF-β; TRAIL/TNFSF10	CTACK/CCL27; GRO-α/CXCL1; MCP-3/CCL7; MIG/CXCL9;SDF-1α/CXCL12	HGF; M-CSF/CSF1;β-NGF; SCGF-β/CLC11
**27-plex (BioRad)**	IL-1β; IL-1Ra; IL-2; IL-4; IL-5;IL-6; IL-9; IL-10; IL-12 (p70);IL-13; IL-15; IL-17; IFN-γ; TNF-α	IL-8/CXCL8; Eotaxin/CCL11;MCP-1/CCL2; IP-10/CXCL10;MIP-1α/CCL3;MIP-1β/CCL4; RANTES/CCL5	IL-7; basic FGF;b;G-CSF; PDGF-BB; VEGF;GM-CSF

Abbreviations: IL-1α, Interleukin 1 subunit α; IL-2Ra, Interleukin 2 receptor alpha; IL-3, Interleukin 3; IL-12p40, Interleukin 12 subunit p40; IL-16 Interleukin 16; IL-18 Interleukin 18; IFN-α2, Interferon alpha 2; LIF, Leukemia inhibitory factor; MIF, Macrophage migration Inhibitory factor; SCF, Stem cell factor; TNF-β, Tumor necrosis factor-b; TRAIL, TNF-related apoptosis inducing ligand; CTACK, Cutaneous T cell-attracting chemokine (CCL27); GROα, Growth-related oncogene alpha; MCP-3, Monocyte chemotactic protein 3 (CCL-7); MIG, Monokine induced by interferon gamma (CXCL9); SDF-1α, Stromal-cell derived factor alpha 1; HGF, Human growth factor; M-CSF, Macrophage-colony-stimulating factor; β-NGF, Nerve growth factor beta; SCGF-β, Stem cell factor growth factor beta; IL-1β, Interlukin 1 subunit β; IL-1Ra, Interleukin 1 receptor alpha; IL-2, Interleukin 2; IL-4, Interleukin 4; IL-5, Interleukin 5; IL-6, Interleukin 6; IL-9, Interleukin 9; IL-10, Interleukin 10; IL-12p70, Interleukin-12 subunit p70; IL-13, Interleukin 13; IL-15, Interleukin 15; IL-17, Interleukin 17; IFN-γ, Interferon gamma; TNF-α, Tumor necrosis factor alfa; IL-8, Interleukin 8; Eotaxin, eosinophil chemotactic protein; MCP-1, monocyte chemoattractant protein1 (CCL2); MIP-1α, Macrophage inflammatory proteins 1 subunit α (CCL3); IP-10, 10 kDa interferon gamma-induced protein (CXCL10); MIP-1β, Macrophage inflammatory proteins 1 subunit β (CCL4); RANTES, regulated on activation T cell expressed and secreted (CCL5); IL-7, Interleukin 7; FGF basic, Basic fibroblast growth factor; G-CSF, Granulocyte colony-stimulating factor; PDGF-bb, Platelet derived growth factor; VEGF, Vascular endothelial growth factor; GM-CSF, Granulocyte-Macrophage Colony Stimulating.

In brief, 50 μL of cervico-vaginal fluid, this amount falling within the linear range of the assay, and standards were added in duplicate to a 96 multiwells plate containing analyte beads. After incubation for 30 minutes at room temperature and washing, the antibody-biotin reporter was added and incubated for 10 minutes with streptavidin-phycoerythrin. The concentrations of the cytokines were determined using the Bio-Plex array reader (Luminex, Austin, TX). The Bio-Plex Manager software optimized the standard curves automatically and returned the data as Median Fluorescence Intensity (MFI) and concentration (pg/mL). This assay has a reported limit of detection of 1–20 pg/ml, depending on the cytokine target [37].

### Statistical analysis

The softwares Stata (v. 13.1) and GraphPad Prism (v. 5) were used for statistical data analysis. For homogeneity of presentation, the values of the cytokines were expressed as medians and interquartile ranges (IQRs). Comparisons between two groups were made using the t-test and the Mann-Whitney test. The Kruskall-Wallis one-way analysis of variance was used to compare more than two groups. When a significant p-value was found (p<0.05), a multiple comparison test was used to determine which groups were different.

## Results

### Association of High-Risk HPV with Cytological Characteristic

A total of 168 women (mean age 30 (SD±4) years) who fulfilled the baseline demographic and microbiologic criteria were enrolled in the study. The correlations of HPV infection and genotype distribution with the type of cervical lesion are described in Table 2. In this series, 47.6% of women were diagnosed with no cervical cytological abnormalities (NIL), 25.5% were diagnosed with LSIL and 26.7% with HSIL. The overall prevalence of HR-HPV as single infection was 66% (112/168). According to the cytological reports, the frequency of infection was 30% in NIL and of 100% in LSIL and HSIL women. HPV-16 was the most frequent genotype (34.4% of all infections) with frequency peaking in women with HSIL (55.5%), followed by genotypes HPV-31 (11.7%) and HPV-33 (8.4%).

**Table 2:** Prevalence of HPV genotypes distribution according to cytological characteristics.

Cytology	n°	HPV-	HR-HPV+	n°/Genotypes
**NEG**	80	56 (70%)	24 (30%)	5/HPV-33
				4/HPV-53°
				3/HPV-31
				3/HPV-66°
				3/HPV-58
				2/HPV-16
				2/HPV-45
				1/HPV-51
				1/HPV-18
LSIL*	43	0 (-)	43 (100%)	14/HPV-16
				7/HPV-53
				7/HPV-31
				6/HPV-66
				3/HPV-18
				2/HPV-58
				1/HPV-35
				1/HPV-73
				1/HPV-56
				1/HPV-39
**HSIL**^§^	45	0 (-)	45 (100%)	25/HPV-16
				6/HPV-18
				5/HPV-33
				4/HPV-31
				2/HPV-52
				2/HPV-51
				1/HPV-45
**TOTAL**	168	56 (32%)	112 (66%)	—

Abbreviation: HPV, human papillomavirus

**°** HPV: genotypes associated with an intermediate oncogenic risk for Cervical Cancer

* LSIL: low squamous intraepithelial lesions

^**§**^ HSIL: high squamous intraepithelial lesions

### Cytokines profile and HR-HPV infection

A panel of 48 soluble immune proteins was measured in the cervical fluids for the evaluation of their expression profile in response to HR-HPV infection. The cytokine profiles of HPV-positive samples were compared to HPV-negative samples. A pattern of 22 molecules was found to be statistically associated with HR-HPV infection (p<0.001) while the remaining 26 did not differ significantly. The concentration of the molecules, measured as pg/ml, and the degree of statistical significance are reported in Table 3. Nineteen cytokines, including angiogenic growth factors and cell proliferation factors, were found to be significantly high in HR-HPV infected women, and showed a distinct pathway probably orchestrated by an epithelial- located HR-HPV activity. Among these factors, the highest values were recorded for CCL7 (MCP-3), IL-12p70, IL-18 and CCL27 (CTACK). Conversely, a decreased release of the pro-inflammatory interleukin15 and interferon (IFN-γ), and of the anti-inflammatory interleukin9, was observed in the same group. The down regulation of these molecules, which are key components for fighting virus infection, prevents the growth and the activity of macrophages, monocytes and T cells and, most importantly, inhibits their immunostimulatory and immunomodulatory effects.

**Table 3:** Concentrations of immune mediators with deregulated expression in cervical-vaginal fluid of HPV positive and HPV negative women.

Immune mediators	HPV—women	HPV + women	Folds of changes
**Pro-inflammatory**			
IL-6***	0(0–1.5)	1.9(0–4.7)	1.9
**IL-12p70** ***	3.8(0–10.8)	15.3(4.9–36.7)	4.0
CCL2-(MCP-1)***	3(2.3–6)	7.2(3.7–14.8)	2.4
CCL-7 (MCP-3)***	2.9(1.7–7.8)	15(2.9–41.7)	5.1
**CXCL9-(MIG)** ***	124(8.5–419.2)	228(28.5–2709)	1.8
G-CSF**	22(7.1–57.4)	55.2(7.6–356.5)	2.5
IL-3***	41.6(29.1–50.6)	93.2(41.1–128.3)	2.2
IL-12p40***	145(60.2–204.5)	329(99.3–657)	2.2
IL-16***	5.5(3.8–10.4)	18(6.6–51.5)	3.2
**IL-18** ***	8.1(4.4–23.7)	35.5(5.5–308)	4.3
**M-CSF** ***	19.4(11.2–45.3)	45.8(17.6–143)	2.3
**TNF-β** ***	0	2.3(0–4)	2.3
**GROα** **	57.9(21.7–432.7)	195(71.8–2969	3.3
**IFN-α**_2_***	9.2(0–10.8)	30.7(9.6–41.4)	3.3
FGF-b**	15(5.3–29.2)	23.3(13.1–34.1)	1.5
**CXCL27 (SDF-1α)** ***	25.6(17.7–138.6)	76.6(36.9–316.9)	3.0
VEGF***	23.1(11.1–60.5)	76.1(35.6–126)	3.2
**CCL27-(CTACK)** ***	0(0–6)	5.2(3.6–9.1)	5.2
IFNγ***	8.8(3.1–15.1)	1(0–10.4)	8.8↓
IL-15***	5.3(2.6–11)	0(0–4.7)	5.3↓
**Anti-inflammatory**			
LIF***	12.7(11.4–16.9)	18.6(12.5–28.8)	1.4
IL-9***	3(2.3–4.3)	0.5(0–2.3)	6.0↓

**^↓^** Down-regulated cytokines

Data are given as medians (quartiles) (pg/ml). The comparison between the two groups was made using the Mann-Whitney non-parametric Student’s t-test. The significant results are marked with asterisks:

**p<0.01

***p<0.001.

### Cytokines profile and HR-HPV- cytological lesions

**Fig 1:**
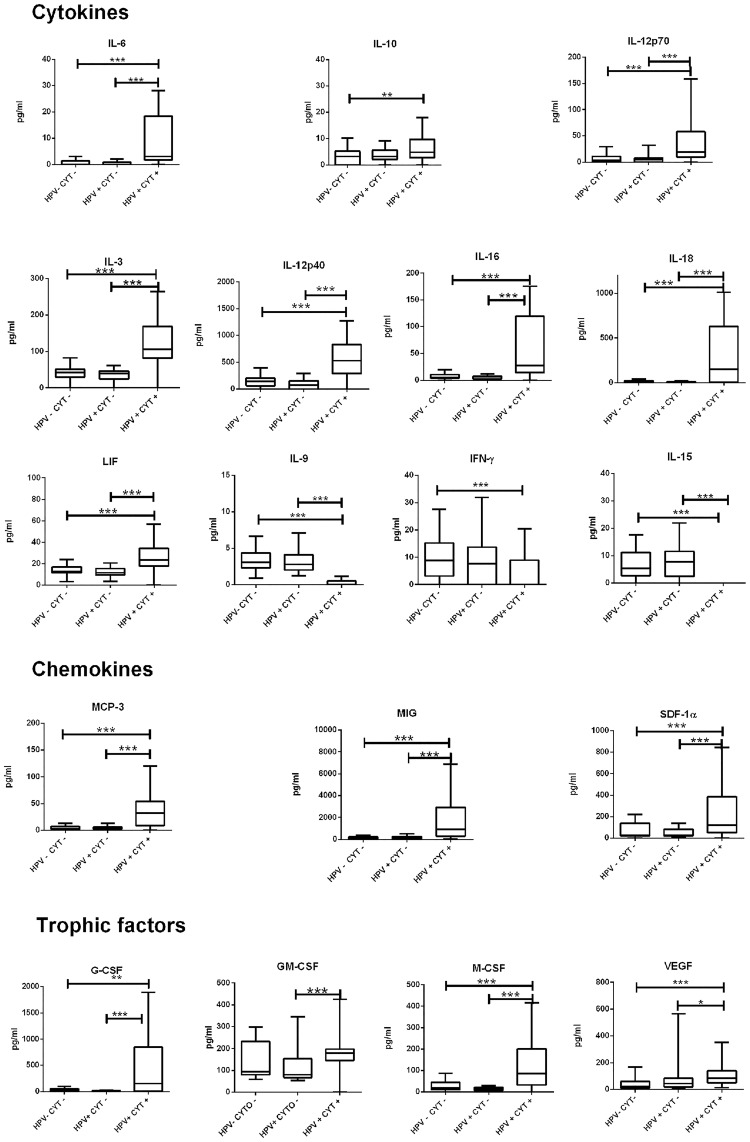
Expression of significant immune mediators in cervical fluid of HR-HPV positive women. The median values of the specific protein found down and up regulated in the local microenvironment of HR-HPV positive women without and with cervical lesions compared to the control group. The box plots show the expression levels (ρg/mL) of the cytokines found to be statistically significant between the study groups. Data expressed as median values are reported in Table 4. The significant results are marked with asterisks: *p<0.05, **p<0.01, ***p<0.001.

Fig 1 shows the deregulated expression of cytokines and their significant association with cervical lesions, regardless of the grade of the lesions. The interleukins IL-6, IL-3, IL-12p40, IL-12p70, IL-16, IL-18, LIF, chemokines SDF-1α, CCL7 (MCP-3), CXCL9 (MIG), the tropic factors VEGF, M-CSF and G-CSF, which were significantly associated with HPV infection (Table 3), showed increased levels in women with cervical neoplasia, regardless of the severity of the lesions, compared to HPV-positive women without cytological abnormalities (HPV+/CYTO-) or to HPV-negative women without cytological lesions (HPV-/CYTO-). Conversely, the concentrations of interleukins IL-9, IL-15 and interferon IFN-γ were lower in the presence of cytological lesions. The median values of these immune mediators are reported in Table 4. IL-10, which was not statistically associated to HPV infection, displayed significantly high levels only in women with HPV-related lesions, compared to HPV-/CYTO- women. These findings confirm the potential role of this interleukin in inducing cervical immunosuppression and profound peripheral tolerance via T-reg cells, and in promoting the development of cytological lesions [37]. As shown Fig 1, no immune mediator previously found associated to viral infection (Table 3), showed a statistical significance between the HPV-/CYT- and HPV+/CYT- groups. In keeping with previous studies, in our series, the “HPV(+) normal cytology” state is presumably consistent with early/latent infection. Only in the presence of cytological damage, we observe a significant disregulation of the expression of some cytokines (e.g: IL-3, IL-16, IL-18, IL-6, MCP-3, etc) [8, 38].

**Table 4:** The median values of significant immune mediators in cervical fluid of HR-HPV positive women.

IMMUNE MEDIATORS	HPV-/CYT- (median value)	HPV+/CYT- (median value)	HPV+/CYT+ (median value)
IL-6	2.30 ρg/mL	1.80 ρg/mL	7.69 ρg/mL
IL-10	3.15 ρg/mL	3.26 ρg/mL	4.76 ρg/mL
IL-12p70	3.84 ρg/mL	5.10 ρg/mL	19.52 ρg/mL
IL-3	41.60 ρg/mL	39.14 ρg/mL	105.4 ρg/mL
IL-12p40	145.5 ρg/mL	72.67 ρg/mL	527.2 ρg/mL
IL-16	5.50 ρg/mL	6.39 ρg/mL	27.69 ρg/mL
IL-18	8.12 ρg/mL	4.03 ρg/mL	146 ρg/mL
LIF	12.72 ρg/mL	11.47 ρg/mL	23.78 ρg/mL
IL-9	3.12 ρg/mL	2.8 ρg/mL	0 ρg/mL
IFN-γ	8.82 ρg/mL	7.61 ρg/mL	0 ρg/mL
IL-15	5.39 ρg/mL	7.65 ρg/mL	0 ρg/mL
MCP-3	2.89 ρg/mL	3.33 ρg/mL	32.46 ρg/mL
MIG	64.79 ρg/mL	114 ρg/mL	921.8 ρg/mL
SDF-1α	25.60 ρg/mL	24.80 ρg/mL	121.9 ρg/mL
G-CSF	22.04 ρg/mL	10.91 ρg/mL	156.1 ρg/mL
GM-CSF	94.94 ρg/mL	90 ρg/mL	180 ρg/mL
M-CSF	19.21 ρg/mL	14.16 ρg/mL	85.09 ρg/mL
VEGF	23.14 ρg/mL	44.99 ρg/mL	85.20 ρg/mL

**Fig 2:**
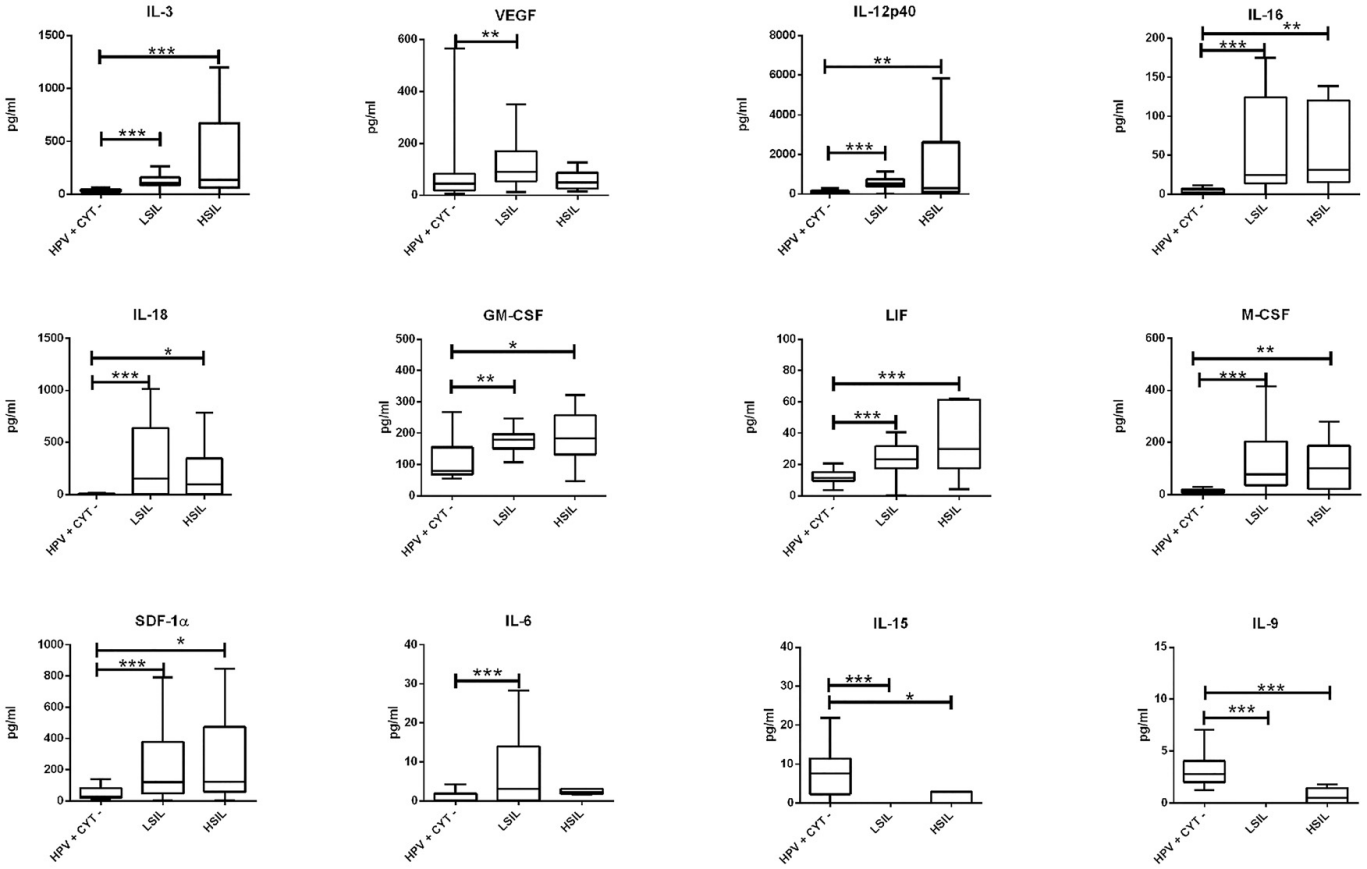
Immune mediators up and down–regulated by HR-HPV infection and severity of induced precancerous lesions (LSIL *vs* HSIL). The box plots show the expression levels (ρg/mL) of the cytokines found to be statistically significant between HPV+CYT- *vs* LSIL *vs* HSIL groups. Data expressed as median values are reported in Table 5. The significant results are marked with asterisks: *p<0.05, **p<0.01, ***p<0.001.

It is worth noting that the growth factor GM-CSF was the only cytokine to be significantly associated with an altered cytology (p<0.001), with concentrations that were 2 times higher (180 pg/mL *vs* 90 pg/mL) than those found in HPV-/CYTO- women. When the severity of the precancerous cervical lesions (LSIL *vs* HSIL) was factored into the analysis (Fig 2), the correlation results indicated the same expression profile identified with the univariate analysis (Fig 1). An increasing gradient of concentration from LSIL to HSIL was measured for IL-16, LIF, CXCL12 (SDF-1α), M-CSF, and GM-CSF, IL-3, but without statistical significance. The respective median values are reported in Table 5.

**Table 5:** The median values of significant immune mediators between HPV+CYT- *vs* LSIL *vs* HSIL groups.

IMMUNE MEDIATORS	HPV+/CYT- (median value)	LSIL (median value)	HSIL (median value)
IL-3	39.14 ρg/mL	104.8 ρg/mL	135.7 ρg/mL
VEGF	44.99 ρg/mL	90.42 ρg/mL	50.09 ρg/mL
IL-12p40	72.67 ρg/mL	539.9 ρg/mL	291.1 ρg/mL
IL-16	6.39 ρg/mL	25.27 ρg/mL	31.68 ρg/mL
IL-18	4.03 ρg/mL	153.5 ρg/mL	99.51 ρg/mL
GM-CSF	90 ρg/mL	178.3 ρg/mL	181.8 ρg/mL
LIF	11.47 ρg/mL	23.36 ρg/mL	29.99 ρg/mL
M-CSF	14.16 ρg/mL	78.26 ρg/mL	101.1 ρg/mL
SDF-1α	24.80 ρg/mL	118.5 ρg/mL	121.9 ρg/mL
IL-6	1.80 ρg/mL	5.95 ρg/mL	4.91 ρg/mL
IL-15	7.65 ρg/mL	0 ρg/mL	0 ρg/mL
IL-9	2.80 ρg/mL	0 ρg/mL	0.50 ρg/mL

## Discussion

During the early stages of the productive infection by human papillomavirus, the innate immune system creates an inflammatory network by recruiting innate immune cells. The activation of keratinocytes Toll-like receptors (TLRs) promotes the production of cytokines, including interleukins IL-1β, IL-6, IL-8, IL12, Tumor necrosis factor (TNFα) and interferons (IFNs), and creates a powerful pro-inflammatory environment [11]. However, HPV seems to be able to modify cytokine expression as an immune evasion mechanism, mainly directed to down-regulating the pro-inflammatory response. There is ample evidence supporting the view that host immunological features and a local HPV- induced immunosuppressive environment constitute key events in persistent infection and development of cervical neoplasia [38,39,40].

Although several studies have described the pleiotropic role of soluble factors in driving the progression of cervical intraepithelial lesions [34], the in vivo analysis of the immune profile in response to HR-HPV infection, prior to the emergence of cellular damage and in the absence of additional risk co-factors affecting the immune pathway, has been little explored.

In the current study, a number of cytokines and growth factors were found to be significantly associated with HR-HPV infection, regardless of the presence of altered cytology (Table 3). In keeping with previous studies, in our series, the “HPV(+) normal cytology” state was presumably consistent with early/latent infection. During this phase, no cytolysis or cytopathic death of keratinocytes occurred as a consequence of virus replication and assembly and,for most of the duration of the HPV infectious cycle, there appears to be little or no release of proinflammatory cytokines. Indeed, the virus is practically invisible to host defenses, which may remain ignorant of its presence for long periods of time [8,38]. According, in the present study a significant disregulation of the expression of some cytokines and growth factors was detected only in presence of cytological damage (Fig 1).

A significant negative modulation was observed for cytokines usually involved in the recruitment and activation of immune cells and key factors during the resolution of an active HPV infection: a down-regulation of IL-9, IL -15 and IFN-γ was observed in all groups included in the study, regardless of the severity of cytological lesions. Growth factors promoting angiogenesis and cell proliferation were found to be over-expressed in infected women and the highest levels of concentration of these factors were measured in women with a cervical lesion. In the present study, the growth factor VEGF, one of the most extensively investigated molecules for its potent pro-angiogenic and mitogenic effect on vascular endothelium in vivo, was found to be significantly over-expressed in women with HPV infection and in women with abnormal cytology but not statistically associated with the severity of the cervical lesions (LSIL *vs* HSIL). A possible explanation could be that the majority of the studies involved this mediator were focused on cervical cancer. Conversely, our study examined the pre-cancer phase including only intraepithelial lesions of low and high grade. During this stage, HPV infected cells have not crossed the basal barrier and appear to be orchestrated by a pattern of induced mediators which are different from those observed in CC [41,42]. This finding seems to be confirmed by the increased production of E6/E oncogenic proteins which are able to trigger a cascade of events leading to the alteration of cell morphology and the production of soluble factors that sustain the tumoral transformation of epithelial cells.

Consistent with previous studies, documenting the ability of the Granulocyte Macrophage Colony-Stimulating Factors (GM-CSF) to induce the migration of antigen presentation cells and cytotoxic T-lymphocytes into tumor lesions [37,43,44], in the current study, GM-CSF was found to be significantly associated with cervical lesions but not with HPV, suggesting an important role for this host immune-mediator in development and progression of the lesion.

The novelty of this study consists in the “in vivo” demonstration of the ability of HR-HPV to drive specific molecules of the local inflammatory response towards cell transformation. This process seems to occur independently of the capacity of the transformed-cells to evade the host immune response through molecules that are thought to deregulate the immune response in several tumors, as recently suggested for CXCL12 (SDF-1α) [13,45]. In the present study, CXCL12 was found to be significantly linked with HR-HPV infection, and increased levels of concentration of this factor have been detected in women with pre-cancerous lesions.

Recent data have suggested that the progression of HPV pre-cancerous lesions depend on both the suppression of cellular immunity, driven by the Th1 response, and the development of the immunosuppressive T-reg profile for neoplastic progression [46,47]. In addition, high levels of pro- inflammatory IL-6 and of anti-inflammatory cytokines, in particular IL-10, TGF-β1 and TGF-β2, have been detected in cervical secretions from LSIL women [15,32,34]. Moreover, Torres-Poveda et al. recently showed that IL-10 has a potent immunosuppressive role in the HPV immune evasion, favoring virus persistence and progression of HPV-related lesions [39]. Several studies have described a pleotrophic multifunction of IL-6, both in chronic inflammation and in some types of cancer, including cervical cancer [48,49]. A possible role for IL-6 in the progression of cervical lesions has already been observed in the human basal cell carcinoma (BCC-1/KMC) cell line. In this model, IL-6 was found to be regulated by the level of expression of the viral E6 and E7 oncoproteins [50]. Consistent with this finding, in our study a significant increase in the expression of IL-6 and IL-10 was observed in the presence of cytological lesions (Fig 1), while no statistical significance was observed for IL-10 when the severity of the lesions was included in the analysis (p>0.05). Notably, the increased expression of IL-6 was associated only with low grade lesions (Fig 2).

In conclusion, data from this “in vivo” study demonstrate that HR-HPV influenced the production of specific inflammatory and growth factors which are involved in early inflammatory response, suggesting that they might trigger transformation and immortalization of epithelial cells, thereby establishing the conditions for the onset of cancer.
